# Accurate, fast, data efficient and interpretable glaucoma diagnosis with automated spatial analysis of the whole cup to disc profile

**DOI:** 10.1371/journal.pone.0209409

**Published:** 2019-01-10

**Authors:** Ian J. C. MacCormick, Bryan M. Williams, Yalin Zheng, Kun Li, Baidaa Al-Bander, Silvester Czanner, Rob Cheeseman, Colin E. Willoughby, Emery N. Brown, George L. Spaeth, Gabriela Czanner

**Affiliations:** 1 Department of Eye & Vision Science, Institute of Ageing and Chronic Disease, University of Liverpool, Liverpool, United Kingdom; 2 Centre for Clinical Brain Sciences, University of Edinburgh, Chancellor's Building, Edinburgh, United Kingdom; 3 St Paul’s Eye Unit, Royal Liverpool University Hospitals NHS Trust, Liverpool, United Kingdom; 4 Medical Information Engineering Department, Taishan Medical School, TaiAn City, ShanDong Province, China; 5 Department of Electrical Engineering and Electronics, University of Liverpool, Brownlow Hill, Liverpool, United Kingdom; 6 School of Computing, Mathematics and Digital Technology, Faculty of Science and Engineering, Manchester Metropolitan University, Manchester, Manchester, United Kingdom; 7 Biomedical Sciences Research Institute, Faculty of Life & Health Sciences, Ulster University, Coleraine, Northern Ireland; 8 Department of Ophthalmology, Royal Victoria Hospital, Belfast, Northern Ireland; 9 Institute for Medical Engineering and Science, Massachusetts Institute of Technology, Cambridge, Massachusetts, United States of America; 10 Anesthesia, Critical Care and Pain Medicine, Massachusetts General Hospital, Harvard Medical School, Boston, Massachusetts, United States of America; 11 Glaucoma Research Center, Wills Eye Hospital, Philadelphia, Pennsylvania, United States of America; 12 Department of Applied Mathematics, Faculty of Engineering and Technology, Liverpool John Moores University, Liverpool, United Kingdom; Bascom Palmer Eye Institute, UNITED STATES

## Abstract

**Background:**

Glaucoma is the leading cause of irreversible blindness worldwide. It is a heterogeneous group of conditions with a common optic neuropathy and associated loss of peripheral vision. Both over and under-diagnosis carry high costs in terms of healthcare spending and preventable blindness. The characteristic clinical feature of glaucoma is asymmetrical optic nerve rim narrowing, which is difficult for humans to quantify reliably. Strategies to improve and automate optic disc assessment are therefore needed to prevent sight loss.

**Methods:**

We developed a novel glaucoma detection algorithm that segments and analyses colour photographs to quantify optic nerve rim consistency around the whole disc at 15-degree intervals. This provides a profile of the cup/disc ratio, in contrast to the vertical cup/disc ratio in common use. We introduce a spatial probabilistic model, to account for the optic nerve shape, we then use this model to derive a disc deformation index and a decision rule for glaucoma. We tested our algorithm on two separate image datasets (ORIGA and RIM-ONE).

**Results:**

The spatial algorithm accurately distinguished glaucomatous and healthy discs on internal and external validation (AUROC 99.6% and 91.0% respectively). It achieves this using a dataset 100-times smaller than that required for deep learning algorithms, is flexible to the type of cup and disc segmentation (automated or semi-automated), utilises images with missing data, and is correlated with the disc size (p = 0.02) and the rim-to-disc at the narrowest rim (p<0.001, in external validation).

**Discussion:**

The spatial probabilistic algorithm is highly accurate, highly data efficient and it extends to any imaging hardware in which the boundaries of cup and disc can be segmented, thus making the algorithm particularly applicable to research into disease mechanisms, and also glaucoma screening in low resource settings.

## Introduction

Glaucoma is a heterogeneous group of conditions with characteristic narrowing of the optic nerve rim and associated loss of peripheral vision. It is the leading cause of irreversible blindness worldwide and its prevalence increases with age. The projected number of people with glaucoma worldwide is estimated to reach 111.8 million in 2040, with the majority of patients living in Asia and Africa [1]. In the UK, approximately 80% of referrals to the hospital eye service originate from routine sight tests by optometrists in the primary eye-care service. However, only about 33% and 38% of routine suspect glaucoma referrals are subsequently found to have glaucoma in the UK [2] and Ireland respectively [3].

Manual detection of glaucoma is a difficult task for humans. Glaucoma is often slowly progressive and difficult to diagnose in the early stages, when treatment to delay progression is most effective. Healthcare systems must therefore accurately distinguish between patients with and without early disease from a large population at risk, using subtle clinical signs. Both over and under-diagnosis have costly implications in terms of treatment and loss of vision [4]. One strategy to address this is the use of virtual clinics, where a clinician reviews test data without personally seeing patients [5]. These can increase the efficiency of medical staff time, but the interpretation of optic disc images and visual field tests still relies on the subjective assessment of a limited number of parameters, which can lead to errors [6]. Similar issues around test interpretation apply to clinical trials of glaucoma treatments and population screening based on disc photography [7]. The Disc Damage Likelihood Scale (DDLS) is probably the most accurate system for manual grading of glaucomatous disc changes, which assesses rim width with reference to disc size and is correlated with visual field loss [8]. However many clinicians continue to measure only the vertical cup/disc ratio, which is attractive for its simplicity and speed, but is a poor marker of glaucoma. All of these points highlight the need for an automated method to assess the optic disc.

Automated approaches to glaucoma have been studied intensively in the last decade with variable success. The simpler machine learning algorithms analyse the vertical cup/disc ratio (vCDR) yielding maximal diagnostic accuracy of 84% [9], and none quantifies the shape of the whole neuro-retinal rim. Deep learning has recently been used to achieve very accurate glaucoma detection [10], albeit with a very large training dataset (n = 31,745) and after removal of a significant number of images deemed unsuitable for analysis (8,371). In other words it is highly accurate but also demanded a large amount of high quality training data.

However, as with other retinal features [11], the shape of the of cup and disc, the location and distribution of optic nerve rim narrowing is likely to be biologically meaningful, as is recognised to some extent in the DDLS [8]. With this in mind, we hypothesised that incorporating a novel model of optic rim width shape could lead to high data efficiency and high generalisation. For this purpose, a hierarchical probabilistic model (aka generative model in deep learning literature, see e.g. [12]) provides a natural framework for inference and discrimination, with high computational speed. The structure of the hierarchical model was determined so that it reflects the geometrical underpinnings of the shape of optic cup and discs so that accurate inference and prediction is possible [13].

Our emphasis on quantifying the shape of the optic nerve rim is in contrast to methods focussing on prediction (such as deep learning) [10], where the biological reasons for accurate discrimination are inherently obscure. Although explanatory models such as ours are not necessarily the best predictive models, both disease explanation and accurate prediction can co-exist, and when this is the case the predictive power helps to justify prior assumptions about disease mechanism [13].

We describe a method for quantifying the shape of the optic nerve, and then use this information to accurately distinguish images of glaucomatous and healthy optic discs with very little data. It works in two steps. First, the disc and cup are segmented and the cup/disc ratio (CDR) is measured in 24 cross-sections to create a cup/disc ratio profile (pCDR). Then, in the second step, the shape of pCDR is analysed using a hierarchical probabilistic spatial model. The spatial model is then used to derive a disc deformation index and a glaucoma detection rule using recent advancements in empirical Bayes predictive methods [14] [15]. Our spatial algorithm has the same accuracy as the modern deep learning algorithm [10] when applied to publicly available datasets (ORIGA and RIM-ONE) which have clinical glaucoma diagnosis as the reference standard [16] [17]. The detection rule reflects the degree to which a given pCDR is more akin to the typical overall shape of a glaucomatous or healthy optic nerve, and we correlate this risk estimate with an automated version of the DDLS.

## Results

### Datasets

To illustrate and test our method, we analysed the ORIGA and RIM-ONE datasets (see Methods). The ORIGA dataset contains 650 retinal fundus images from subjects with or without glaucoma (n = 149 and 501 respectively) [16]. RIM-ONE consists of 159 images from subjects classed as glaucoma positive, negative, or glaucoma suspect (n = 39, 85 and 35, respectively) [17]. Both image datasets have semi-automated disc segmentation data. We also performed our own automated image segmentation (see Methods) to indicate the boundary of the disc and of the cup.

### Cup/disc ratio profile (pCDR)

**Fig 1:**
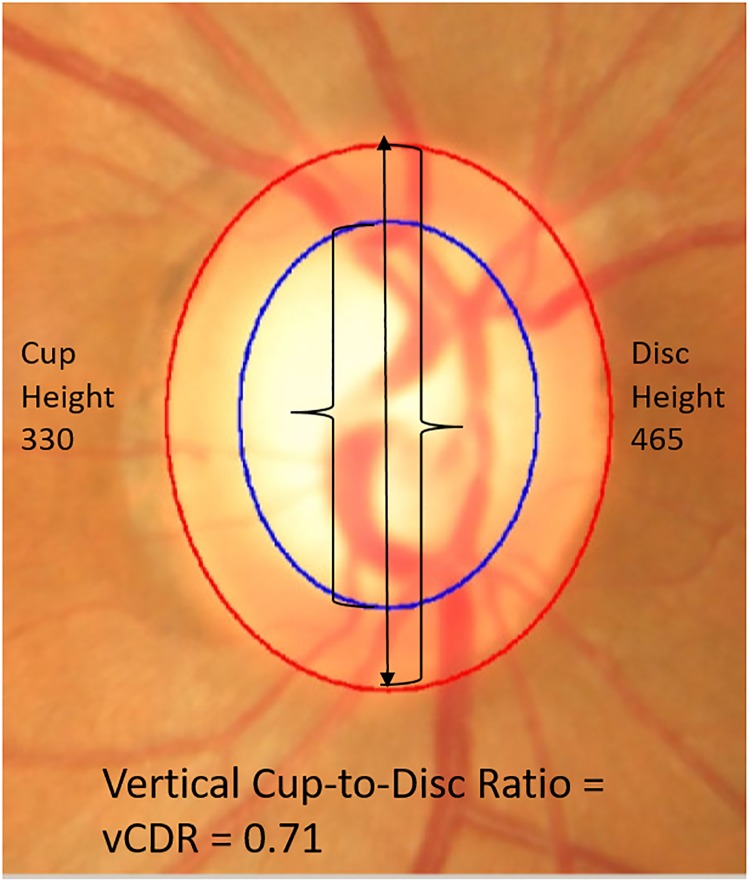
Example of vertical cup/disc ratio. Here, the boundaries of the cup and disc were determined using the ORIGA-GT software (modified from [16]). This software generates boundaries by fitting two ellipses using human expert landmark identification and least squares fitting. The cup boundary is given in blue; the disc boundary is in red. In the text, this is referred to as semi-automated segmentation.

**Fig 2:**
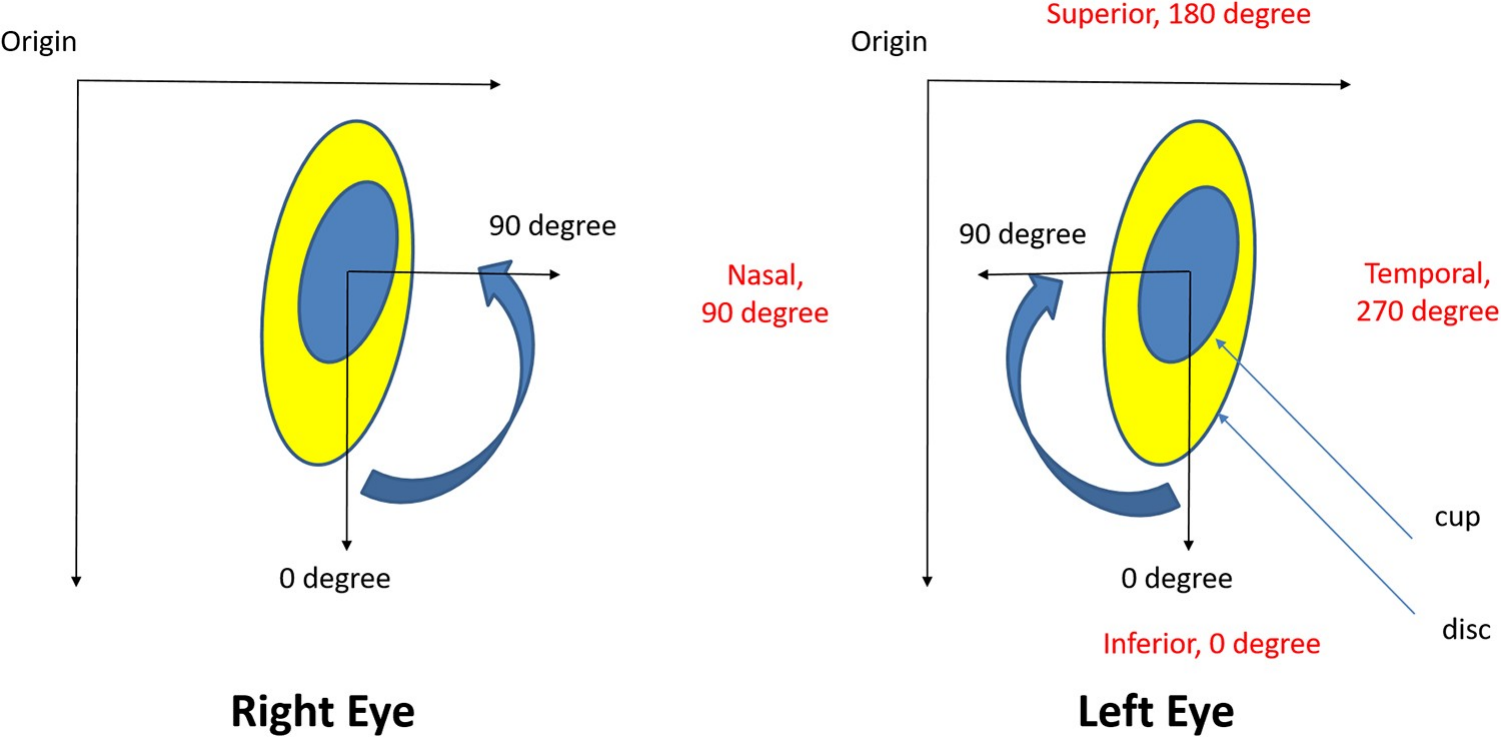
Orientation of the landmarks in the right and the left eye. The centre of the cup is used for the calculations.

Traditionally, assessment of optic nerve rim width is only carried out in the vertical meridian, yielding the vertical cup to disc ratio, vCDR (Fig 1). However, glaucomatous optic neuropathy can affect the nerve rim at any point and this characteristic is not captured well by measuring the CDR in only one meridian. Therefore, in order to increase the accuracy of glaucoma detection, we calculated 24 CDR values around the whole cup and disc at 15-degree intervals. We thus created a CDR profile (pCDR), which is a vector of these 24 values. In order to be consistent, the vector direction was indexed clockwise for left eyes and anti-clockwise for right eyes (Fig 2).

**Fig 3:**
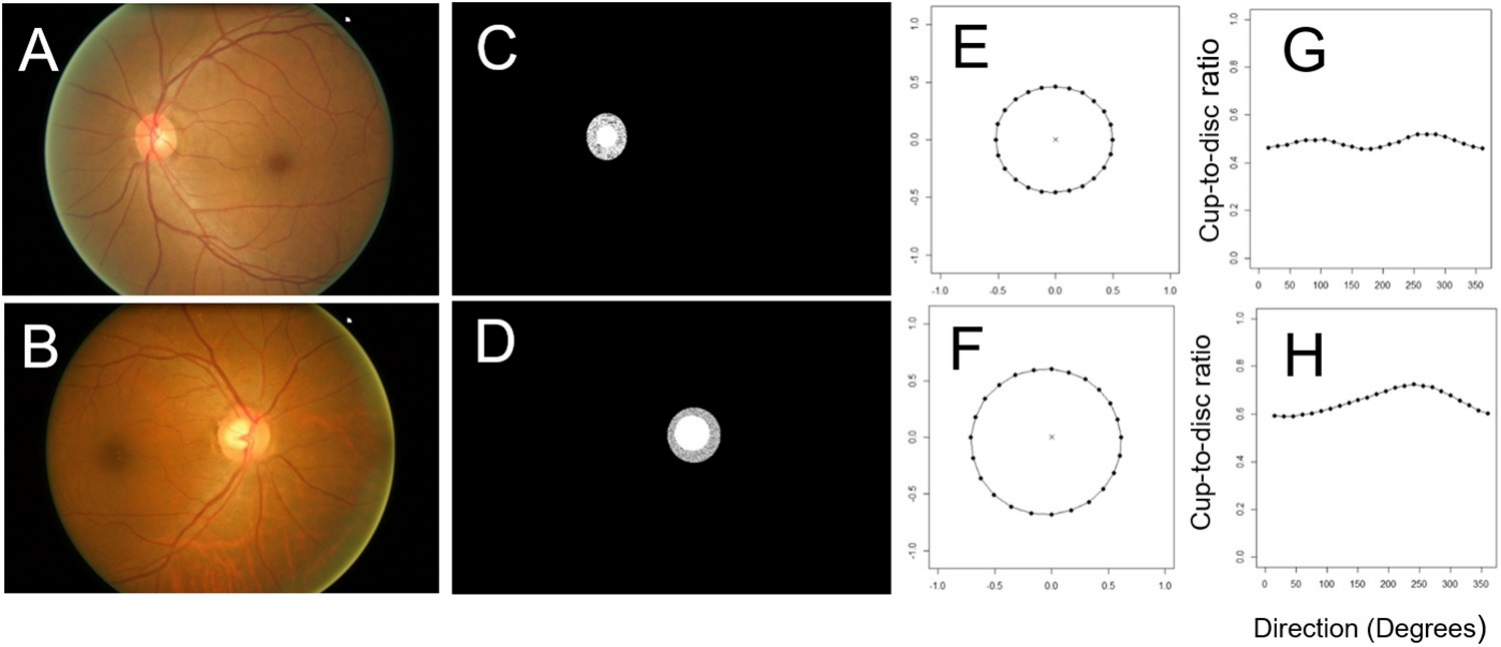
The profile of 24 cup/disc ratios (pCDR) in two eyes. One healthy fundus (A) and one glaucomatous fundus image (B) are showed here. The cup and disc were semi-automatically segmented, which is shown by the best-fitting ellipses (C and D). The profile of 24 CDR values were plotted in circular (E and F) and Cartesian systems (G and H).

In both datasets, for each optic nerve image, we created a spatially resolved pCDR (Fig 3). This consists of 24 numbers between 0 and 1 which can be plotted on a circular (Fig 3E and 3F) or Cartesian system (Fig 3G and 3H) to allow visual interpretation of the deformations.

### The shape of the optic nerve head in healthy and glaucomatous cases

**Fig 4:**
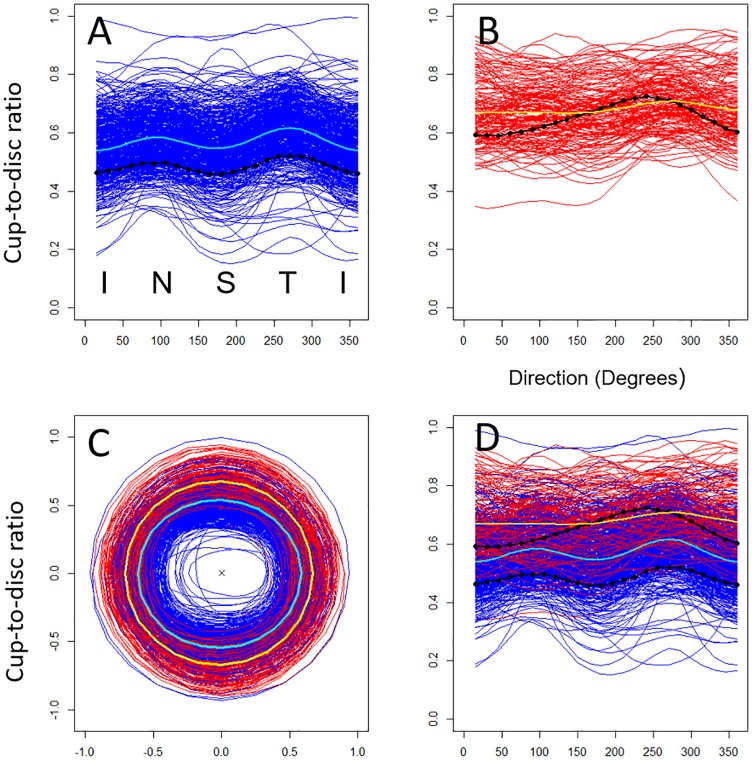
The cup/disc ratio profiles (pCDR) of all individual eyes from ORIGA. Individual healthy (A) and glaucomatous (B) optic nerve images from the ORIGA dataset (n = 650) in circular (C) and Cartesian (D) formats. These profiles come from semi-automated segmentation. The population mean pCDR for healthy (cyan) and glaucomatous (yellow) groups are shown together with the individual pCDR profiles of the two eyes from Fig 3 (black).

There is a large overlap in pCDR between the healthy and glaucomatous optic nerves (Fig 4, blue vs red). The mean pCDR of the healthy optic discs shows two peaks with maximum CDR at 90 and 270 degrees (Fig 4A and 4D, cyan). This profile appears to be consistent with the ISNT rule, which states that in healthy discs the rim is typically widest (i.e. lowest CDR) inferiorly, then superiorly, then nasally, and finally temporally [18]. The individual pCDR profiles show large variability around this mean profile, owing to inter-subject differences in the size of the disc—a factor not normally included in CDR models. In contrast, although inter-individual variability is present, the mean pCDR profile for glaucomatous discs is notably flatter compared to that of healthy eyes, with generally greater cup-to-disc ratios (Fig 4B, yellow). Individual glaucomatous pCDR profiles generally appear to break the ISNT rule (Fig 3H).

To characterise the observed differences of pCDR profiles between healthy and glaucomatous eyes formally, we fitted a probabilistic spatial model to the pCDR profiles in all ORIGA images (Methods) which uses goniometric functions to describe the shape of the pCDRs. As observed in the plot (Fig 4), the model confirms that the population pCDR profiles are not constant on a Cartesian system (i.e. not a circle in a circular system) (Table 1, Direction, p-value<0.001); the two disease groups differ in terms of the pCDR mean (Table 1, Overall group effect, p-value<0.001) as well as the shape of the pCDR (Table 1, Direction*Group, p-value<0.001). The population mean pCDR profiles calculated from the spatial model coincide with the raw mean profiles (S1 Fig), indicating that the spatial model is a good fit to the data. This analysis quantifies the shape characteristic of the CDR in healthy and glaucomatous eyes that had previously only been described semi-quantitatively in systems such as the Disc Damage Likelihood Scale [6] [8]. This proves that there is a significant difference in shape between glaucomatous and healthy discs and that these differences are in all 24 directions, not just in the vertical direction. The spatial model of pCDR allows these subtle differences between healthy and glaucomatous discs to be quantified. In what follows, we show how we used the spatial model to derive a glaucoma detection algorithm.

**Table 1:** Fitted spatial statistical model and association with disease group in the ORIGA dataset.

Associations using all images and using statistical spatial model of CDR profile		Num df	Den df	F Statistic	P-value
Source of variation					
Fixed effects	Intercept	1	14946	29068.881	<0.001
	Direction	4	14946	3295.685	<0.001
	Overall group effect	1	648	189.723	<0.001
	Direction x Group	4	14946	461.653	<0.001
Random effect	Between eye variation, SD	0.0892			
Random term	Within subject variation, SD	0.0414			
Spatial correlation	Modelled via random effect	0.8227			

The cup and disc data used here come from semi-automatic segmentation. Test statistics (F Statistic and P-value) for the associations of individual components of the model are given together with the degrees of freedom for the numerator and denominator (Num df and Den df).

### Principle assumptions of the glaucoma detection algorithm

We built our detection algorithm on four key assumptions. Assumption 1: a manual, semi-automated or automated segmentation of the cup and disc is possible and therefore one can produce a pCDR for each eye (Methods, see details of segmentation). Assumption 2: the deformation of the glaucomatous optic nerve head manifests into a change in the shape of the pCDR profile. This assumption is confirmed in Fig 4 and Table 1. Assumption 3: the healthy optic nerve head has a shape that can be approximated by two ellipses. Assumption 4: the size of the optic disc can differ across subjects owing to factors such as genetics. To this end, we progressively built our framework by characterising variations in the pCDR profiles for the healthy and glaucomatous optic nerve heads in one spatial probabilistic model and then used it to derive the diagnostic decision rule.

### The algorithm estimates the probability of glaucoma for a given pCDR profile

The diagnosis of a new eye proceeds by first obtaining the pCDR profile of its optic nerve head, ***Y***_*new*_, and by calculating the posterior probability, *p*_*new*,*G*_, of being glaucomatous using Bayes theorem:

$$p_{new,G}=\frac{p_{G}f_{G}\left(Y_{new}\;|\hat{\beta },\hat{V}\right)}{p_{G}f_{G}\left(Y_{new}\;|\;\hat{\beta },\hat{V}\right)+p_{H}f_{H}\left(Y_{new}\;|\;\hat{\beta },\hat{V}\right)},$$ (1)

where $f_{G}(Y_{new\;}|\hat{\beta },{\hat{\sigma }}_{d}^{2},\;{\hat{\sigma }}_{e}^{2})$ and **$f_{H}(Y_{new}\;|\;\hat{\beta },{\hat{\sigma }}_{d}^{2},\;{\hat{\sigma }}_{e}^{2})$** are the multivariate normal probability density functions with means $X_{G}\hat{\beta }$ and $X_{H}\hat{\beta }$, respectively; and common variance-covariance matrix $\hat{V}$ (see Methods). The matrices ***X***_*G*_ and ***X***_*H*_ are design matrices incorporating the direction (angle) and identifiers of the groups. The values of the vector $\hat{\beta }$ and matrix $\hat{V}$ are obtained via restricted maximum likelihood by fitting the spatial model to the training dataset of images (see Methods).

### The proposed diagnostic decision rule for the spatial detection algorithm

The probabilities, *p*_*H*_ and *p*_*G*_, in Eq (1) are the prior probabilities of the eye being healthy and glaucomatous, respectively, and can be estimated using the observed proportions of optic discs in the data. The posterior probability in Eq (1) was derived using the empirical Bayes predictive method [14] [15] [19] [20] and using the estimated spatial probabilistic model. The posterior probability of the new eye belonging to the healthy group can be calculated analogically to Eq (1) or it can be simply obtained as *p*_*new*,*H*_ = 1 − *p*_*new*,*G*_.

The posterior probability in Eq (1) can be used to propose a glaucoma detection rule. The simplest detection rule is to compare this posterior probability with a predefined probability threshold, *p*_*th*_:

$$\begin{array}{r} If\;p_{new,G}\geq p_{th},conclude\;that\;the\;eye\;is\;glaucomatous,\\ If\;p_{new,G}<p_{th},conclude\;that\;the\;eye\;is\;healthy \end{array}$$ (2)

**Fig 5:**
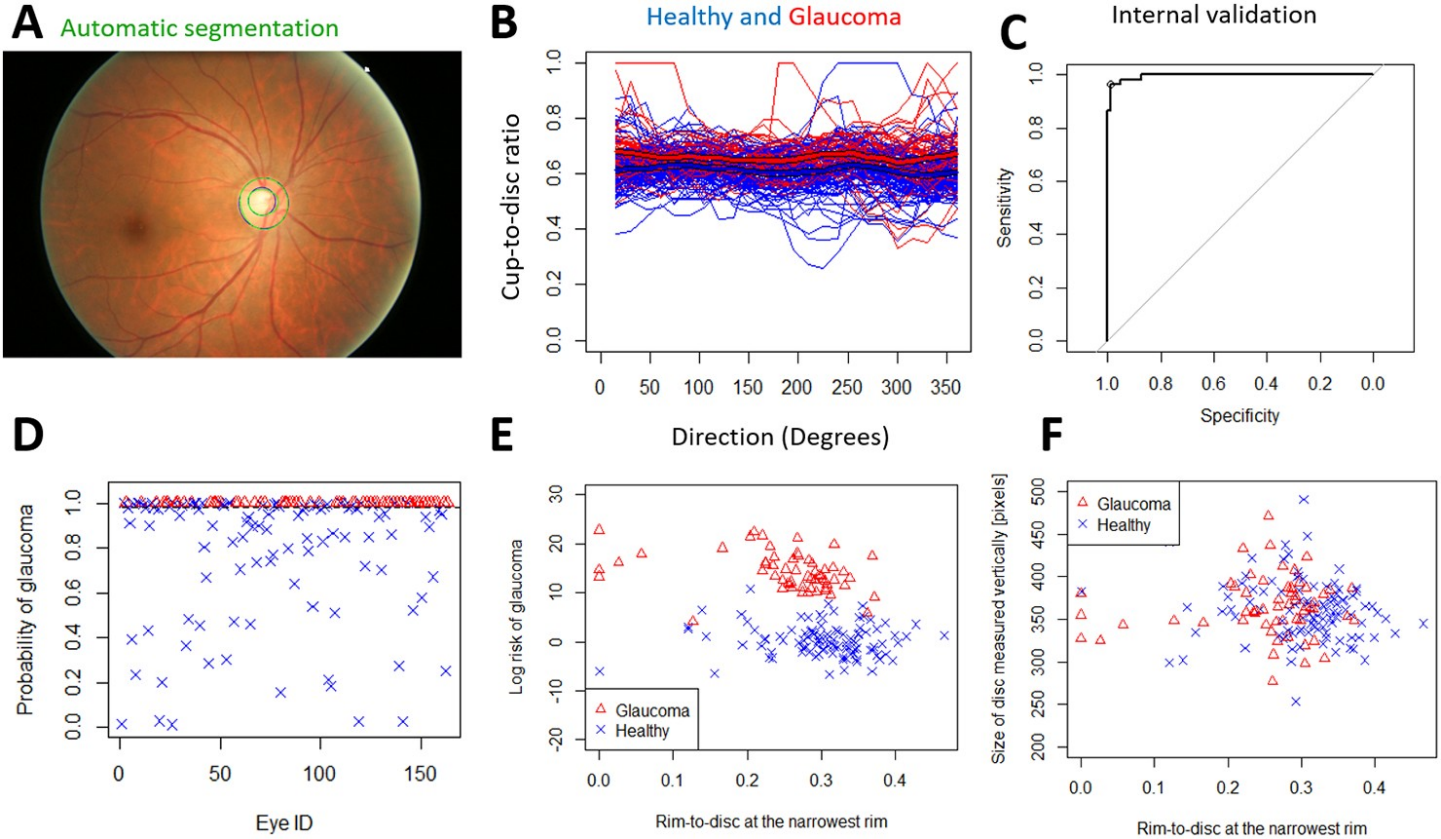
Internal validation of the spatial algorithm using automatically segmented images from ORIGA. A) The grader's semi-automatic segmentation (blue) and the fully automatic segmentation (green). B) The individual automatically segmented profiles with means (thick blue line for healthy, thick red line for glaucomatous). We used the automatically segmented discs and cups to detect glaucoma. C) The AUROC is 99.6%. D) The probability of glaucoma and the decision threshold for 96.6% sensitivity and 99.0% specificity. The size of the testing dataset is n = 163. E) The risk of glaucoma (*log*(*p*/(1 − *p*))) vs Rim-to-Disc at the narrowest rim. F) The Rim-to-Disc at the narrowest rim vs disc size.

There are several strategies for selecting the threshold probability, *p*_*th*_. One strategy is to choose *p*_*th*_ that corresponds to the point closest to the top left hand corner of the receiver operating characteristic (ROC) curve (Fig 5C) thus yielding a so-called optimal threshold that minimises the overall misclassification. Another strategy is to follow a clinical objective. For instance, if the detection rule (Eq 2) is used for screening, then the priority is to minimise false negatives. This could be achieved by decreasing the threshold.

It is important to note that the detection rule in Eq (2) has an intuitive interpretation. By construction, the log odds of the glaucoma (Eq 1) is equal to the difference of two Mahalanobis distances, the new disc from the typical healthy profile, and the new disc from the typical glaucomatous profile, hence the log-odds can be interpreted as a Disc Deformation Index (see Methods). Consequently, the detection rule in Eq (2) yields the diagnostic decision based on *the shape* of the pCDR (i.e. the presence and number of pCDR peaks) rather than on the difference of pCDR from the typical pCDR of healthy or glaucomatous discs (i.e. vertical separation on the y-axis in Fig 4). This is because the rule (Eq 2) is based on the posterior probability, *p*_*new*,*G*_, which provides an absolute measure of risk for the optic disc whose pCDR is equal to ***Y***_*new*_. Since this probability is calculated from the parameters of the spatial model, this probability doesn’t reflect *raw* differences of ***Y***_*new*_ from mean glaucomatous and healthy pCDR, ***X***_*G*_***β*** and ***X***_*H*_***β***, but rather *covariance-rescaled* differences which is effectively a shape comparison [21]. In summary, the probability (Eq 2) quantifies whether the shape of a new optic nerve image is more likely to be similar to that of a glaucomatous or healthy nerve.

Consequently, if a new eye has a small but healthy disc then all its measured pCDR values are shifted up by some number—i.e. the measurements are higher than the typical profile of glaucomatous discs (Fig 4D, yellow). The proposed algorithm indirectly takes into account the size of the optic disc. Clinically, the size of the optic disc has been shown to be important to the detection of glaucoma [8], for example, a given rim width (e.g. CDR 0.7) may be normal in a large disc, but indicate disease in a small disc. Indeed, in the dataset we observed that if an healthy disc has both a large CDR and a large disc height then all its measured pCDR values are shifted up by some number (Fig 4D, top blue profiles), and therefore might appear to be glaucomatous (at least, in euclidian terms) even though it is not. To correct for this we do not need to know the value of the constant that shifts the profile up or down. Instead, we assume that such a number exists and that it can be modelled by an optic disc specific random effect within the spatial probabilistic model. This allows our method to solve the problems with classification arising from high inter-individual variation in disc size, without relying on an absolute measure of disc height. Estimation with cup/disc ratios rather than microns or pixels has the advantage that the probability estimate does not require correction for image magnification—which varies between cameras, and indeed, eyes.

### Performance of the spatial detection algorithm in internal validation with semi-automatic segmentation

First, we evaluated the glaucoma detection algorithm on the ORIGA dataset with internal validation, usingsemi-automated optic disc segmentation. The diagnostic rule based on a 24-dimensional pCDR (Eq 2) yielded almost perfect detection (AUROC = 99.7%, S2 Fig) with 100% sensitivity and 98.3% specificity (S2 Fig), in internal validation and using semi-automated segmentation. This represents a 15.7% improvement on the existing detection algorithms that use vertical CDR (AUROC = 84% [9]), and results from two points. This large improvement is a combination of two phenomena: using whole profiles rather than the vertical CDR in isolation improves the classification from 84% to 88% AUROC; and adjusting for spatial correlations within each profile (using random effects, hence adjusting for disc size) leads to a further 11.7% improvement, from 88% to 99.7%.

### The spatial detection algorithm compared with support vector machine (SVM) learning analysis of the pCDR

To further validate our detection algorithm, we compared it with SVM in the internal validation of 100 bootstrapped samples. Each time, we split the ORIGA dataset randomly into 70% training data and 30% testing data (Table 2). For each split we calculated the accuracy of our spatial statistical algorithm and SVM, both using the 24-dimensional pCDR. The accuracy of the spatial detection algorithm was substantially higher (mean AUROC 98.3%, range 85.1% to 99.7%) when compared to SVM (AUROC 82.4%, range 76.1% to 88.0%).

**Table 2:** Comparison of the spatial algorithm with machine learning (SVM) for the classification of glaucoma.

	Spatial algorithm	SVM
**Average AUROC [%]**	98.3	82.4
**Standard deviation AUROC [%]**	3.1	2.3
**Minimum AUROC [%]**	85.1	76.1
**Maximum AUROC [%]**	99.7	88.0
**Average sensitivity [%]**	95.4	74.3
**Average specificity [%]**	94.2	79.3

We used 100 random splits of the ORIGA dataset (70% training, 30% testing). Both the spatial algorithm and SVM used the full pCDR, rather than the simple vertical vCDR alone. These data comes from semi-automated segmentation.

### Performance of the spatial detection algorithm in internal validation with automatic segmentation

Next, we aimed to see how well the detection algorithm works if the disc and cup segmentation is fully automated, rather than using a semi-automated method. We used the semi-automated segmentation as the ground truth to train our automated segmentation algorithm. 75% of the ORIGA dataset and the corresponding semi-automatically segmented optic heads were used to train the automatic segmentation (Methods) and to train the glaucoma detection method. We then applied automated segmentation to the remaining 25% of images (n = 163) (Fig 5A). This resulted in a larger overlap between disease groups (Fig 5B). However, the healthy optic nerve heads still clearly showed similar population profiles with two humps with distance of 180 degrees (Fig 5B). As with semi-automated segmentation, the glaucomatous optic heads appear to show a flatter average profile (Fig 5B). We then used the trained glaucoma detection algorithm (trained on 75%, semi-automatically segmented data) to detect glaucoma on the 25% automatically segmented images. The final AUROC was 99.6% (Fig 5C), 96.6% sensitivity and 99.0% specificity and with clear separation of healthy and glaucomatous discs (Fig 5D).

### Performance of the spatial detection algorithm in external validation with semi-automated segmentation

We tested our rule (Eq 2 fitted to the ORIGA dataset) using semi-automatically segmented optic nerves from the RIM-ONE dataset as a means of external validation and obtained an AUROC of 89.9% (S3 Fig).

### Performance of the spatial detection algorithm in external validation with automated segmentation

**Fig 6:**
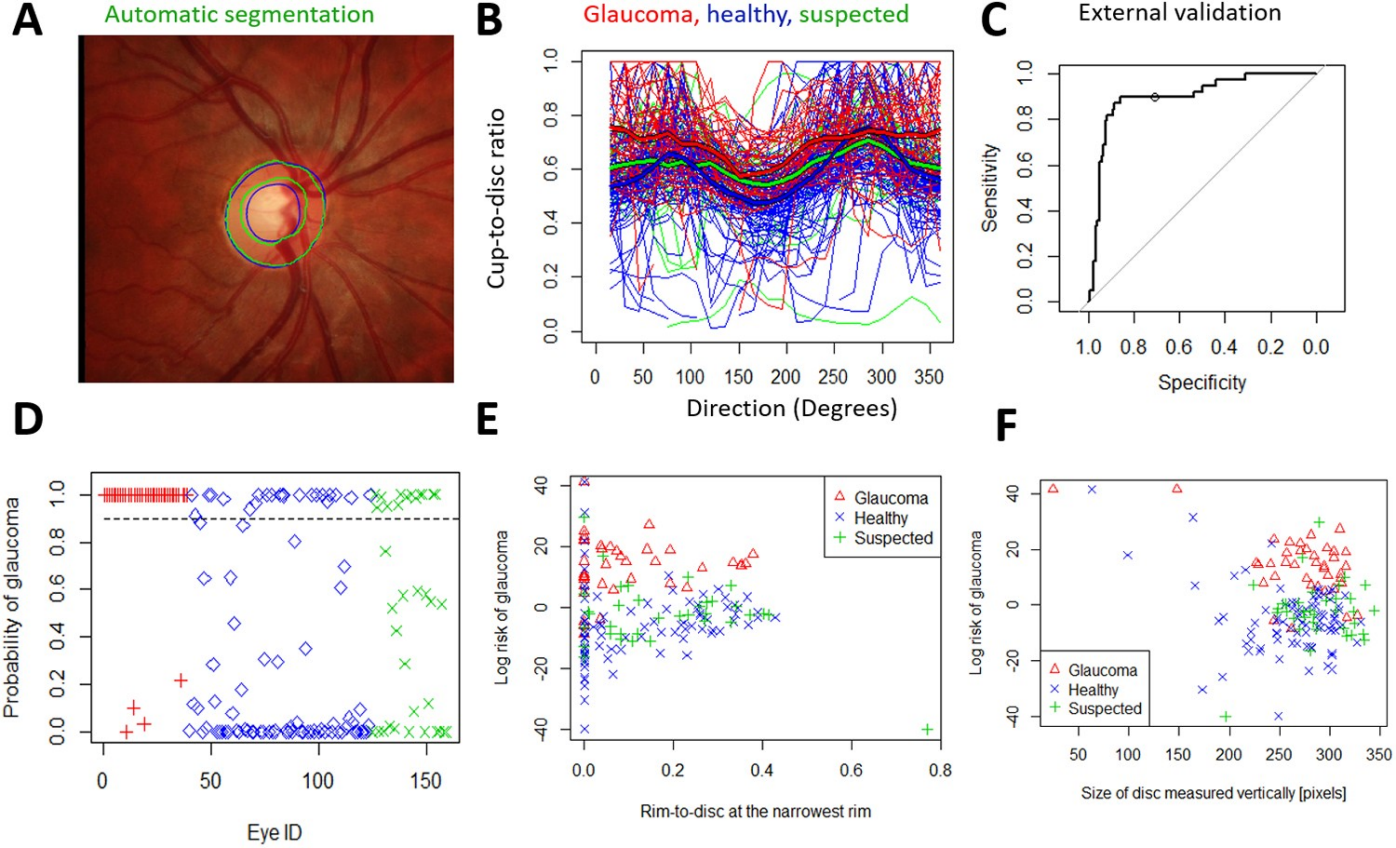
External validation of the spatial detection algorithm using the automatically segmented images from RIM-ONE. Here, all ORIGA-light images were used to train the segmentation and the glaucoma detection. The RIM-ONE images were then automatically segmented and glaucoma detection was tested. A) The grader's semi-automatic segmentation (blue) and the fully automatic segmentation (green). B) The individual automatically segmented profiles of 39 glaucomatous, 85 healthy and 35 suspected optic discs. C) The AUROC in external validation was 91.0% for discrimination between glaucomatous and healthy. The threshold probability of 0.90 (see the circle) yields 89.7% sensitivity and 74.1% specificity. D) The posterior probability of glaucoma in the three RIM-ONE groups with the 0.90 threshold (dashed line). The algorithm identified as glaucomatous: 35 out of 39 glaucomatous (90%), 22 out of 85 healthy (26%), and 13 out of 35 suspected (37%) eyes. E) The risk of glaucoma (log(p/(1-p)) vs Rim-to-Disc ratio at the narrowest rim. F) The risk of glaucoma vs disc size.

We aimed to see how the spatial algorithm performs when a training dataset (ORIGA) is used for both training the segmentation algorithm and to derive the glaucoma detection rule (Eq 2). The testing dataset for the glaucoma detection was the RIM-ONE dataset. We found excellent accuracy (AUROC 91.0%) (Fig 6A–6C). The posterior probability illustrates good separation between groups (Fig 6D and 6E) with the glaucoma suspects having intermediate probabilities. The posterior probability of glaucoma in the three RIM-ONE groups with the 0.90 probability threshold (dashed line). The algorithm identified as glaucomatous: 35 out of 39 glaucomatous (89.7%), 22 out of 85 healthy (26%), and 13 out of 35 glaucoma suspect (37%) eyes.

### Robustness of the spatial algorithm to incomplete disc image data

In some eyes the pCDR profiles were not complete since the segmentation algorithm did not locate the whole boundary of the cup or disc (Fig 6B). However, the hierarchical spatial model is robust to missing profile data and so eyes with incomplete pCDR were fully utilised in the detection algorithm without the need for imputation.

### Comparing the spatial detection algorithm with the Disc Damage Likelihood Scale (DDLS)

Our estimated glaucoma probability (Eq 1) can be related to the DDLS, with which a clinician evaluates the disc height and rim-to-disc ratio at the narrowest area of the rim [6] [8]. We calculated the rim-to-disc ratio at the narrowest point (RTD) (Fig 5, 5E and 5F) and disc size vertically (DSV) (in number of pixels). We assumed consistent magnification of the disc image within each dataset.

The estimated log odds of glaucoma (i.e. the Disc Deformation Index) appeared to increase with smaller RTD (p = 0.02) and DSV (p = 0.08 in unadjusted correlation, p<0.001 in adjusted correlation analysis), in the automatically-segmented images from ORIGA (Fig 5E and 5F), as expected, because glaucoma is more likely with narrowing of the disc rim for a given disc height. In contrast with the results of the spatial algorithm, the combination of DSV and RDT distinguish healthy from glaucomatous with only 74.4% AUROC in ORIGA dataset.

In RIM-ONE automatically-segmented images the estimated log odds of glaucoma (i.e. the Disc Deformation Index) also appeared to give visibly higher discrimination between disease groups (Fig 6E and 6F). It increased with smaller DSV (p = 0.005) and with narrower rim-to-disc ratio (p = 0.05 in unadjusted correlation, p<0.001 in adjusted correlation for the disc size). Our algorithm appeared to give visibly higher discrimination between disease groups (Fig 6E and 6F) while the DSV and RDT can distinguish healthy from glaucomatous with only 61.0% AUROC.

## Discussion

In summary, our spatial model of the optic nerve pCDR discriminates glaucomatous from non-glaucomatous optic discs with high accuracy on internal and external validation (AUROC 99.6% and 91.0% on ORIGA and RIM-ONE images, respectively) with either semi-automated and automated image segmentation; and with high data-efficiency. To the best of our knowledge, this is the first spatial model of the optic disc. Importantly, it explicitly quantifies disc features known to be biologically relevant to glaucoma, and the output is correlated with an existing clinical grading tool (the DDLS). Consequently, the results are applicable to two types of clinical question: firstly, about whether a disc is glaucomatous or not, and secondly *why* the algorithm classified the disc in a certain way.

Disc size is an essential component of the DDLS, since a given CDR may be normal or abnormal depending on the height of the disc. Our spatial model does not incorporate absolute disc height, and as a result does not require factors to correct for variation in image magnification. Instead we model disc size indirectly using a random eye-specific component, and estimate the log odds of glaucoma in terms of a multivariate comparison of a new disc pCDR to reference values. This comparison of Mahalanobis distance interprets each one of the 24 CDR in the context of every other CDR, and allows the model to detect differences in disc shape. It appears that loss of the normal elliptical shape described by the ISNT rule is an important distinguishing feature picked up by the algorithm.

We developed and validated the model on separate image datasets. Our detection accuracy is markedly superior to a recent sparse group lasso method developed on the same ORIGA dataset of 650 eyes (AUROC 84%, in internal validation), which in turn was superior to a list of other methods (AUROC 76% to 84%) (reviewed in [9]). Furthermore our AUROC is comparable to a recent deep learning algorithm (AUROC 98.6% in [10]). Therefore, our spatial detection algorithm represents a significant advance in the automated interpretation of optic disc images.

It also has operational advantages. For example, it can be run quickly on a basic laptop, does not require a very large training dataset. The hierarchical model allows for future additional levels to incorporate information about right and left eyes, and change in the disc profile over time. Formulation in terms of Bayes theorem means that additional glaucoma risk factors (e.g. ethnicity, age, and intra-ocular pressure) can be added easily to the prior probability and frame the analysis of disc shape in a wider clinical context. The ability to detect not only abnormalities at baseline but also subtle changes between clinical visits is particularly valuable in a slowly progressive disease such as glaucoma. Hierarchical models can be run using open source software (e.g. the R package nlme, at https://cran.r-project.org/). We are preparing code for our spatial algorithm for public download from the Liverpool John Moores University webpage and plan to make it part of the R library.

Optimising our method for glaucoma screening (sensitivity and specificity: 96.6% and 99.0% in internal validation) would mean that a significant number of unnecessary hospital visits could be prevented. If we use our results from external validation, and assume 3.5% prevalence in a 100,000 population, 95% sensitivity leads to a reduction of manual testing from 100,000 to 45,785 while 3,325 (out of 3,500) glaucomatous cases would be correctly detected (S1 Table).

There are two main reasons for the high accuracy in glaucoma detection with the presented glaucoma detection algorithm. Firstly, the incorporation of additional biologically relevant information into the model in the form of the pCDR means that estimation is based on a small number of salient parameters. Secondly, our method incorporates variation in optic disc height indirectly *via* random effects. Consequently, our model evaluates disc cupping around the whole disc at 15-degree intervals, and is therefore able to assess asymmetry of the disc within and between patients, while considering other factors in a hierarchical model. Therefore, our model is arguably a method of quantifying and automating semi-quantitative clinical assessment, such as the DDLS [8], which evaluates maximal disc narrowing at any location while taking disc size into account. Human vision relies on specific neurones that detect shapes and edges [22], and in common with clinical assessment, the spatial paradigm moves beyond simple counting of lesion size or frequency, to discernment of lesion location within the context of anatomical symmetry. Similar principles apply to other optic neuropathies with distinctive spatial distributions, such as the “bow-tie” atrophy seen in some cases of chiasmal compression [23].

Spatial modelling allows multiple measures to be analysed simultaneously while accounting for autocorrelations and therefore avoids the problem of multiple comparisons. This advantage is also seen in the analysis of fMRI images using spatial models in contrast to voxel-wise analysis [24].

This approach contrasts with recent developments in deep learning for glaucoma detection, which can achieve very high accuracy after removal of 18% of poor quality images [10]. However deep learning can have disadvantages. These include the need for very large training datasets (30,000 in [10]), and lack of insight into mechanisms underlying disease processes. Our spatial approach has advantages in both of these areas, in uses a training set of approximately 300 images, it can be used independently and it could be used to produce input to a neural network to help overcome sensitivity to missing image data. Indeed, neural networks can be made more data-efficient if they utilize feature contours [12].

### Limitations

We analysed monoscopic images. Although stereoscopic examination may be desirable, monoscopic images are suitable for glaucoma detection [10], and our results show that monoscopic image data can be used effectively to increase diagnostic accuracy.

We used images labelled as glaucomatous or healthy as the derivation dataset (ORIGA). This limits the extent of our analyses since, in clinical practice, many patients are reviewed as glaucoma suspects until diagnosis is clarified over time. An ideal output would quantify both a baseline glaucoma risk and rate of progression, since this would help classify clinically indeterminate cases as well as indicate the need for additional treatment. Further work could be done on prospective cohorts to address this.

Nevertheless, our method performs well on images from publically available datasets ORIGA and RIM-ONE, suggesting it may be of benefit to clinical pathways and population based screening programmes [7]. Many glaucoma studies have relied on the measurement of intra-ocular pressure, even though it is well known that this is a poor marker of glaucoma status [25]. Visual field loss is unquestionably an important clinical outcome in glaucoma, but as a psychophysical measurement, it depends on patient attention as well as overall visual acuity. These are often diminished in the population at risk for glaucoma from co-morbidities such as cognitive impairment and cataract. Consequently, an objective assessment of anatomical changes underlying visual field loss can potentially provide valuable context to the interpretation of other tests in clinical practice and research.

### Conclusion

We present a novel spatial algorithm for assessing glaucoma in images of the optic nerve, along with a method for automated image segmentation. This has several strengths, including high accuracy achieved on derivation and validation datasets. In contrast to predictive strategies involving machine learning (including deep learning), the spatial model provides a Disc Deformation Index that directly reflects clinically relevant features of the optic disc. The method is robust to missing data and extendable to incorporate additional risk factors or image data in extra levels of the hierarchical model or as a prior probability of glaucoma. These features suggest our spatial model is a promising candidate for further development as a diagnostic tool in clinical practice.

## Methods

### Image datasets and patients

To illustrate the new diagnostic framework, we used two large publically available datasets. We were masked to disease status when applying segmentation and running the algorithm. The first dataset consists of retinal fundus images from the Singapore Malay Eye Study (SiMES) [26], a population-based study, which we used to develop the model and discrimination rule. SiMES examined 3,280 Malay adults aged 40 to 80, of which 149 were glaucoma patients. Retinal fundus images of both eyes were taken for each subject in the study. All retinal images were anonymised by removing individually identifiable information before being deposited to the ORIGA-light online database [16]. The investigators then built a database with 650 retinal images including all 168 glaucomatous images and 482 randomly selected non-glaucoma images. There is no description of selection based on image quality [15].

We used a second dataset (RIM-ONE) to externally validate our discriminatory rule. It consists of 159 stereo retinal fundus images with optic disc and cup ground truth [16]. The reference segmentations wereprovided by two experts in ophthalmology from the Hospital Universitario de Canarias. The database comprises healthy patients (n = 85), glaucoma patients (n = 39), and glaucoma suspects (n = 35).

### Data availability, regulations, guidelines and consent of patients

RIM-ONE is a publicly available dataset. In the associated paper [27] the authors state that the study was performed in accordance with the ethical standards laid down in the 1964 Declaration of Helsinki. Approval by the Ethics Committee was obtained and the patients were informed about the study objectives. ORIGA is also a publicly available dataset, a subset of the data from the Singapore Malay Eye Study (SiMES), collected from 2004 to 2007 by the Singapore Eye Research Institute and funded by the National Medical Research Council. All images were anonymised before release.

### Semi-automatic segmentation of optic cup and disc

In the semi-automatic segmentation, an expert grader provides key clinical landmarks along the disc and cup boundary. Then the software ORIGA-GT generates the boundaries by fitting two ellipses, via a least-squares fitting algorithm, yielding two ellipses: one for the cup and one for the disc (Fig 1) [9].

### Automatic segmentation of optic cup and disc

In the automatic segmentation, we find the boundaries of the optic disc (OD) and cup (OC) by training a dense fully convolutional deep learning model on data annotated by an expert grader. This model adapts the DenseNet architecture [28] to a fully-convolutional neural network (FCN) [29] for fully automated OD and OC segmentation [30]. The resulting trained model is used to provide pixel-wise classification of images previously unseen by the model as (i) optic cup, (ii) optic disc rim and (iii) background. This information can then be used to determine the segmentation of the image data, giving the boundaries of the optic disc and cup from which measurements may be taken for Glaucoma diagnosis.

We trained the segmentation model using a set 520 images selected randomly from the ORIGA dataset (80%), of which 130 (25%) are reserved for validation. This trained network is then used to obtain the segmentations of the remaining unseen 130 fundus images. We also test this idea on the whole RIM-ONE dataset by training on the green channel of the 75% ORIGA data (rather than full colour) to improve generalisation and testing this on the green channels of the RIM-ONE images.

For direct comparison with the results of Zhang et al. [16], we split the ORIGA dataset into 50% for training and 50% for testing, which are consistent with sets A and B of [16], respectively.

Finally, for comparison with the expert grader’s segmentation on the entire ORIGA dataset, we aimed to provide an automatic segmentation of the whole ORIGA dataset. Although this is provided by the previous experiment, the significantly reduced training size (80% to 50%) is likely to have significantly adversely affected the results by considerably reducing the training data. To overcome this, we use the idea of k-fold cross validation. That is, we partition the ORIGA dataset into 4 sets ($O_{3}^{1},\ldots ,O_{3}^{4}$) such that the intersection of any two is the empty set. We then carry out four independent tests, by reserving the set $O_{3}^{i}$ for testing and training the network on the remaining 75% of images. Combining the results, we achieve the automatic segmentations of the whole ORIGA dataset.

### The spatial model of the shape of the optic nerve head

In this paper, we propose a spatial model of the 24-dimensional pCDR profile data. The spatial model is in the framework of mixed effects models (e.g. [19] [20] in longitudinal data, [11] in clinical imaging data) also known as hierarchical models.

Let ***Y***_*i*_ = [*Y*_*i*,1_,…,*Y*_*i*,24_]′ be the 24-dimensional response vector for the eye *i*, i.e. pCDR = ***Y***_*i*_, where ***Y***_*i*,*d*_ is the CDR value in direction *d*, *d* = 1,…,24, and where the direction *d* corresponds to the angle *d* × 15° (Fig 2). Then the spatial hierarchical model for eye *i* has the following form

$$Y_{i}=X\beta +Z_{i}d_{i}+e_{i},$$

where ***X*** and ***Z***_*i*_ are matrices of explanatory variables. The matrix ***X*** contains effects of groups (healthy and glaucoma), angle and interaction terms, the matrix ***Z***_*i*_ contains columns for random effects. The parameter vector ***β*** is a *q* × 1 vector of fixed effects regression parameters where *q* is the number of fixed effects parameters. The vector ***d***_*i*_ is a *s* × 1 vector of individual random effects where *s* is the number of random effects. Similarly, the vector ***e***_*i*_ = [*e*_*i*,1_,…,*e*_*i*,24_]′ is the *r* × 1 vector of error terms, where *r* = 24. We assume that ***d***_*i*_~*N*(0,***D***) where ***D*** is a *s* × *s* covariance matrix of random effects and ***e***_*i*_~*N*(0,***R***), and ***d***_*i*_ and ***e***_*i*_ are independent.

In order to find the most parsimonious spatial model, we considered several specifications of the fixed and random effects and we followed the standard model selection procedure (e.g. [19] [20]). First, we found the best specification for fixed effects. To account for the effect of group (glaucoma vs healthy) we included overall means for each group and the indicator functions for the groups. To assure the continuity of pCDR between measurements at consecutive angles we used sine and cosine harmonic functions because they are naturally defined on a circular system. In total, five goniometric functions were considered (e.g. for frequencies 2*πd*/24, …,10*πd*/24) and compared via Bayesian Information Criteria (BIC) and Akaike Information Criteria (AIC). The most suitable order of the harmonic functions turned out to be the second order, which is consistent with the assumption that the shape can be approximated by an ellipse. Furthermore, we added the effect of groups (glaucoma and healthy) and the interactions between group and the goniometric functions because they also decreased AIC and BIC.

Next, we tested several random effect specifications. The only important effect was found to be the overall intercept term for the eye. Such a random effect accounts for the differences in the size of the discs across subjects and it effectively accounts for the spatial correlations. Furthermore, to assess the adequacy of the model, we checked for autocovariance in the residuals by computing the sample variogram (not shown) which indicated that the residuals are uncorrelated. We also computed residuals and plotted them against direction (i.e. the direction from the centre of the optic disc). These residual plots (not shown) did not exhibit any systematic patterns that would give reason for concern over the model fit.

The final best fitting spatial statistical model for pCDR of eye *i* in direction *d* was:

$$\begin{array}{c} Y_{i,d}=\beta_{G,0}I_{G}+\beta_{H,0}I_{H}\\ +\beta_{G,1}sin\left(2\pi d/24\right)I_{G,d}+\beta_{G,2}cos\left(2\pi d/24\right)I_{G,d}\\ +\beta_{G,3}sin\left(4\pi d/24\right)I_{G,d}+\beta_{G,4}cos\left(4\pi d/24\right)I_{G,d}\\ +\beta_{H,1}sin\left(2\pi d/24\right)I_{H,d}+\beta_{H,2}cos\left(2\pi d/24\right)I_{H,d}\\ +\beta_{H,3}sin\left(4\pi d/24\right)I_{H,d}+\beta_{H,4}cos\left(4\pi d/24\right)I_{H,d}\\ +d_{i}+e_{i,d}, \end{array}$$

where

$$[\begin{array}{c} d_{i}\\ e_{i} \end{array}]\sim N(\left[\begin{array}{c} 0\\ 0 \end{array}],[\begin{array}{cc} \sigma_{d}^{2} & 0\\ 0 & \sigma_{e}^{2}I_{24\times 24} \end{array}\right]),$$

and where *β*_*G*,0_ and *β*_*H*,0_ is the intercept for the glaucoma and healthy groups, *I*_*G*_ and *I*_*H*_ are indicator functions for healthy and glaucoma, respectively; and *I*_*G*,*d*_ is an indicator function for the glaucoma group and direction *d*, and *I*_*H*,*d*_ is an indicator function for the healthy group and direction *d*. The best fitting spatial model has 10 fixed effects (*q* = 10), one random effect (*s* = 1), the design matrix of random effects is simply ***Z***_*i*_ = 1 and the random effect vector ***d***_*i*_ is a univariate normally distributed random variable with mean zero and variance $\sigma_{d}^{2}.$ The vector of error terms ***e***_*i*_ has a variance-covariance matrix $R=\sigma_{e}^{2}I_{24\times 24}$.

In the best fitting spatial model of pCDR, the design matrix, ***X***, for the glaucomatous eyes is

$$\begin{array}{c} X_{G}=\left[\begin{array}{cccccccccc} 1 & 0 & sin\left(2\pi \;/24\right) & cos\left(4\pi \;/24\right) & sin\left(2\pi \;/24\right) & cos\left(4\pi \;/24\right) & 0 & 0 & 0 & 0\\ \vdots & \vdots & \vdots & \vdots & \vdots & \vdots & \vdots & \vdots & \vdots & \vdots \\ 1 & 0 & sin\left(48\pi \;/24\right) & cos\left(96\pi \;/24\right) & sin\left(48\pi \;/24\right) & cos\left(96\pi \;/24\right) & 0 & 0 & 0 & 0 \end{array}\right] \end{array},$$

for the healthy eyes is

$$\begin{array}{c} X_{H}=\left[\begin{array}{cccccccccc} 0 & 1 & 0 & 0 & 0 & 0 & sin\left(2\pi \;/24\right) & cos\left(4\pi \;/24\right) & sin\left(2\pi \;/24\right) & cos\left(4\pi \;/24\right)\\ \vdots & \vdots & \vdots & \vdots & \vdots & \vdots & \vdots & \vdots & \vdots & \vdots \\ 0 & 1 & 0 & 0 & 0 & 0 & sin\left(48\pi \;/24\right) & cos\left(96\pi \;/24\right) & sin\left(48\pi \;/24\right) & cos\left(96\pi \;/24\right) \end{array}\right], \end{array}$$

where the 10-dimensional vector of unknown parameters is

$$\beta =[\beta_{G,0},\beta_{H,0},\beta_{G,1},\beta_{G,2},\beta_{G,3},\beta_{G,4},\beta_{H,1},\beta_{H,2},\beta_{H,3},\beta_{H,4}]\prime ,$$

while there are two additional unknown variance parameters, $\sigma_{d}^{2},\sigma_{e}^{2}$.

All these 12 parameters are estimated from all the imaging pCDR data profiles in a single analysis via restricted maximum likelihood procedure lme in R statistical package thus yielding the estimates

$$\hat{\beta }=[{\hat{\beta }}_{G,0},{\hat{\beta }}_{H,0},{\hat{\beta }}_{G,1},{\hat{\beta }}_{G,2},{\hat{\beta }}_{G,3},{\hat{\beta }}_{G,4},{\hat{\beta }}_{H,1},{\hat{\beta }}_{H,2},{\hat{\beta }}_{H,3},{\hat{\beta }}_{H,4}]\prime$$

and

$${\hat{\sigma }}_{d}^{2},\;{\hat{\sigma }}_{e}^{2}.$$

Once the best fitting model and its parameter estimates are found, the marginal distribution in healthy and glaucomatous eyes can be estimated. The marginal distribution for the glaucomatous eye *i* is given by ***Y***_*i*,*G*_~*N*(***X***_*G*_***β***,***V***) and for the healthy eye is given ***Y***_*i*,*H*_~*N*(***X***_*H*_***β***,***V***), where $V=\sigma_{d}^{2}+\sigma_{e}^{2}I_{24\times 24}$ is the marginal covariance matrix for eye *i* (see e.g. [15] [19]).

Then, given the prior probabilities of the diagnostic groups glaucomatous and healthy, *p*_*G*_ and *p*_*H*_, and applying Bayes theorem [13], the posterior probability that the eye *i* with the observed data, pCDR = ***Y***_*i*_, belongs to glaucomatous group is given by

$$p_{i,G}=\frac{p_{G}f_{G}(Y_{i}|\beta ,V)}{p_{G}f_{G}(Y_{i}|\beta ,V)+p_{H}f_{H}(Y_{i}|\beta ,V)},$$

where *f*_*G*_(***Y***_*i*_|***β***,***V***) is the multivariate normal probability density function with mean ***X***_*G*_***β*** and variance-covariance matrix ***V*** and *f*_*H*_(***Y***_*i*_|***β***,***V***) is the multivariate normal probability density function with mean ***X***_*H*_***β*** and variance-covariance matrix ***V***. We note here, that due to the simplicity of the spatial model, the matrix ***V*** is the same for both diagnostic groups. Then, to estimate the posterior probability, *p*_*i*,*G*_, we replaced the unknown parameters with the estimated values of the parameters $\hat{\beta }$ and $\hat{V}={\hat{\sigma }}_{d}^{2}+{\hat{\sigma }}_{e}^{2}I_{24\times 24}$. This posterior probability can be showed to be related to difference in Mahalanobis distances [21]

$$log\frac{p_{i,G}}{1-p_{i,G}}=log\frac{p_{G}}{1-p_{G}}+\frac{1}{2}(D_{H}-D_{G}),$$

where *D*_*H*_ and *D*_*G*_ are Mahalanobis distances between the new data, pCDR = ***Y***_*i*_, and the healthy or glaucomatous group, respectively. Then the difference *D*_*H*_ − *D*_*G*_ can be seen as the disc deformation index: large positive value indicate glaucoma (i.e. *D*_*H*_ > *D*_*G*_, the disc is more similar to glaucoma than healthy disc), large negative values indicate healthy group (i.e. *D*_*H*_ < *D*_*G*_, the disc is more similar to healthy than glaucomatous disc).

## Supporting information

S1 Table Optimisation for glaucoma screening. Each probability threshold value corresponds to one value on the AUROC curve i.e. to one pair of sensitivity and specificity values. Improving the sensitivity necessarily means that the specificity worsens, and vice versa. For example if we choose a threshold probability of 0.90 this leads to sensitivity and specificity of 89.7 and 74.1%, respectively.
(TIF)

S1 Fig The spatial model gives the mean pCDR in each disease group, using semi-automated segmentation data. The population mean profiles calculated from the spatial model coincide well with the raw mean profiles (cyan for healthy, yellow for glaucomatous). Profiles for individual eyes show large between eye variation (blue for healthy, red for glaucomatous).
(TIF)

S2 Fig The internal validation of the glaucoma detection algorithm in ORIGA dataset, using semi-automated segmentation. A) The grader's semi-automated segmentation (blue) was used in this analysis. B) The training set of 325 images was used to fit the spatial model and to derive the parameters of the posterior probability of glaucoma. Then the posterior probability of the glaucoma was calculated for the testing set of 325 images. This posterior probability has AUROC of 99.6% with the optimal threshold at 0.96 (circle at AUROC curve). C) The posterior probability of the testing 325 images and the optimal detection threshold (dashed line). Zero (out of 96) glaucomatous eyes were detected as healthy and 4 (out of 229) healthy eyes were detected as glaucomatous i.e. 100% sensitivity and 93.8% specificity.
(TIF)

S3 Fig External validation of the spatial algorithm in semi-automated RIM-ONE data. The AUROC for discrimination between glaucoma and healthy is 89.9%.
(TIF)
