# Correction: Accurate, fast, data efficient and interpretable glaucoma diagnosis with automated spatial analysis of the whole cup to disc profile

**DOI:** 10.1371/journal.pone.0215056

**Published:** 2019-04-03

**Authors:** Ian J. C. MacCormick, Bryan M. Williams, Yalin Zheng, Kun Li, Baidaa Al-Bander, Silvester Czanner, Rob Cheeseman, Colin E. Willoughby, Emery N. Brown, George L. Spaeth, Gabriela Czanner

The second author, Bryan M. Williams, should also be listed as a corresponding author. Dr. Williams’ email address is: bryan.williams@liverpool.ac.uk.
