# Supplementary figures and images for: Accurate, fast, data efficient and interpretable glaucoma diagnosis with automated spatial analysis of the whole cup to disc profile
Source: PLoS One. 2019 Jan 10;14(1):e0209409. doi: 10.1371/journal.pone.0209409 (PMC6328156; doi:10.1371/journal.pone.0209409)

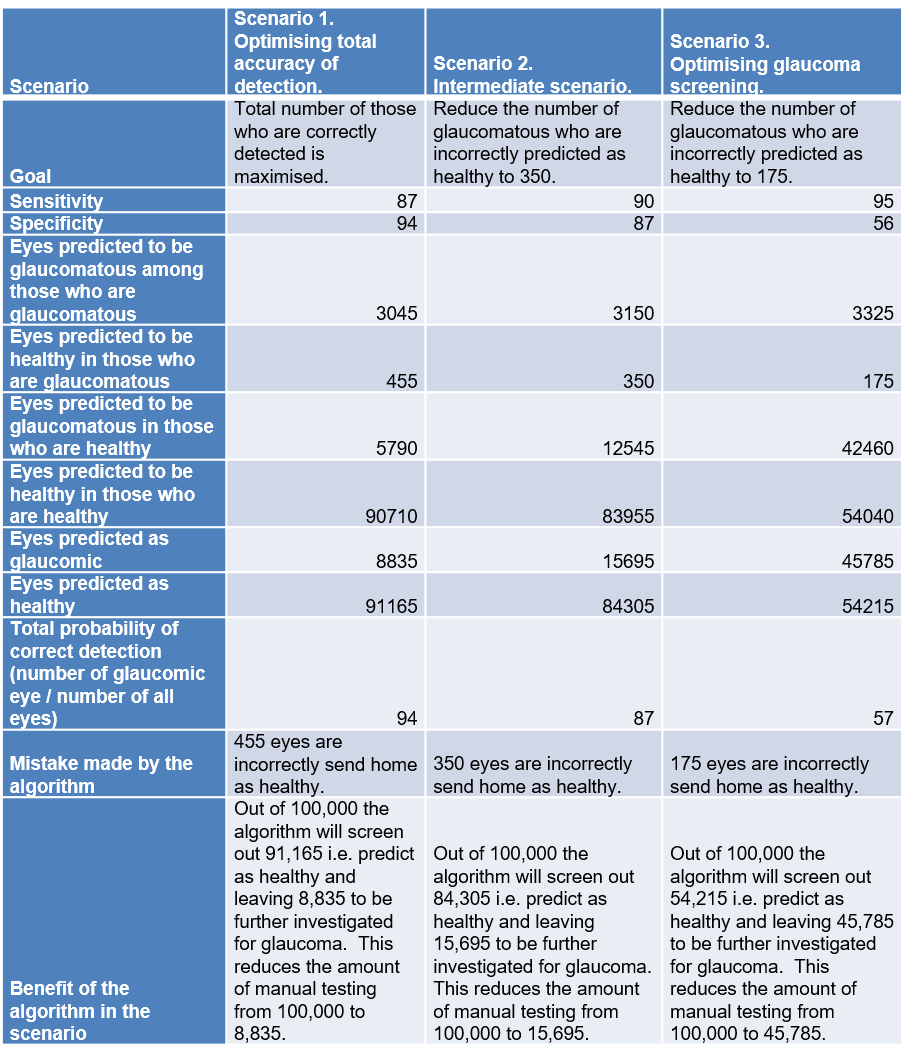

Supplement: S1 Table — Each probability threshold value corresponds to one value on the AUROC curve i.e. to one pair of sensitivity and specificity values. Improving the sensitivity necessarily means that the specificity worsens, and vice versa. For example if we choose a threshold probability of 0.90 this leads to sensitivity and specificity of 89.7 and 74.1%, respectively. (TIF) [file pone.0209409.s001.tif]

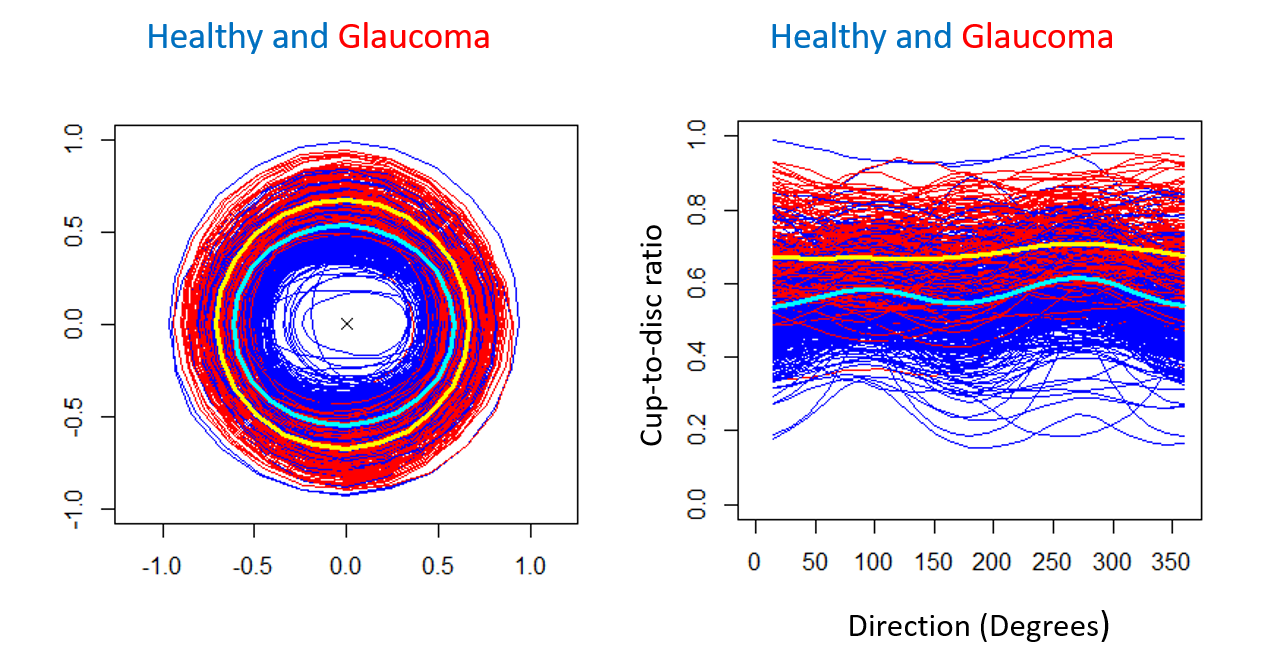

Supplement: S1 Fig — The population mean profiles calculated from the spatial model coincide well with the raw mean profiles (cyan for healthy, yellow for glaucomatous). Profiles for individual eyes show large between eye variation (blue for healthy, red for glaucomatous). (TIF) [file pone.0209409.s002.tif]

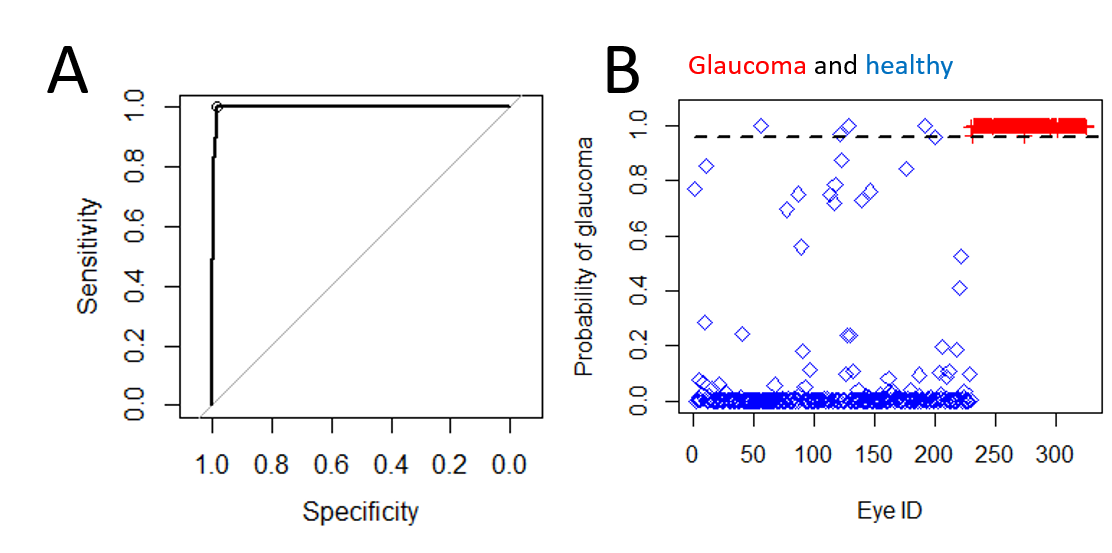

Supplement: S2 Fig — A) The grader's semi-automated segmentation (blue) was used in this analysis. B) The training set of 325 images was used to fit the spatial model and to derive the parameters of the posterior probability of glaucoma. Then the posterior probability of the glaucoma was calculated for the testing set of 325 images. This posterior probability has AUROC of 99.6% with the optimal threshold at 0.96 (circle at AUROC curve). C) The posterior probability of the testing 325 images and the optimal detection threshold (dashed line). Zero (out of 96) glaucomatous eyes were detected as healthy and 4 (out of 229) healthy eyes were detected as glaucomatous i.e. 100% sensitivity and 93.8% specificity. (TIF) [file pone.0209409.s003.tif]

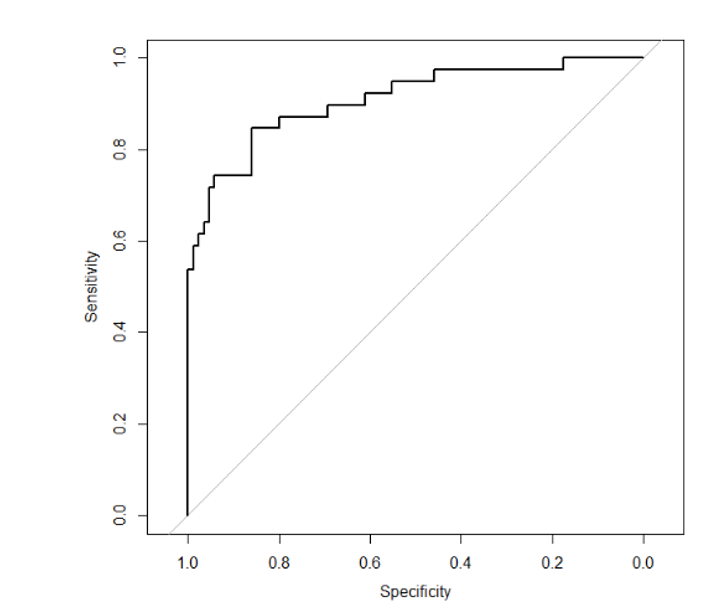

Supplement: S3 Fig — The AUROC for discrimination between glaucoma and healthy is 89.9%. (TIF) [file pone.0209409.s004.tif]
